# Rings of congruence preserving functions

**DOI:** 10.1007/s00605-017-1105-3

**Published:** 2017-11-08

**Authors:** C. J. Maxson, Frederik Saxinger

**Affiliations:** 1 Department of Mathematics, Texas A&M University, College Station, TX 77843-3368 USA; 2 Institute for Algebra, Johannes Kepler Universität-Linz, 4040 Linz, Austria

**Keywords:** Congruence preserving functions, 1-affine complete, Normal subgroup lattice, 08A30, 16Y30, 20E15

## Abstract

Let $C_0 (G)$ denote the near-ring of congruence preserving functions of the group *G*. We investigate the question “When is $C_0 (G)$ a ring?”. We obtain information externally via the lattice structure of the normal subgroups of *G* and internally via structural properties of the group *G*.

## Introduction: background and notation

Let $G=\langle G,+,0\rangle$ be a group, written additively but not necessarily abelian, with neutral element 0. The structure of the near-ring $C_0 (G)=\langle C_0 (G),+,\circ \rangle$ of zero fixing congruence preserving functions on *G* has been the topic of several previous investigations [1, 4]. In this paper we initiate the study of characterizing those groups *G* such that $C_0 (G)$ is a ring.

This investigation also has roots in universal algebra. Recall that a unary polynomial function $p:G\rightarrow G$ is a function that can be written in the form $p(x):=a_0 +k_0 x+a_1 +k_1 x +\cdots +a_{n-1}+k_{n-1}x+a_n ,x\in G, n$ a nonnegative integer, $a_0 ,a_1 ,\dots ,a_n \in G, k_0 ,\dots ,k_{n-1}\in \mathbb {Z}$. We let $P_0 (G)=\langle P_0 (G),+,\circ \rangle$ denote the near-ring of zero preserving polynomial functions on *G* (See [1, 11].)

The near-ring $C_0 (G)$ is a subnear-ring of the near-ring $M_0 (G):=\{f:G\rightarrow G\mid f(0)=0\}$ of zero fixing self maps on *G* where, as usual, the operations in $M_0 (G)$ are pointwise addition of functions and composition of functions. Let $\text {Inn }(G)$ denote the semigroup (under composition) of inner automorphisms of the group *G* and let *I*(*G*) denote the subnear-ring of $M_0 (G)$ generated additively by $\text {Inn }(G)$. From the definitions we see $P_0 (G)=I(G)$.

Now let $f\in C_0 (G)$. Then by definition, for each congruence, $\rho$ of *G*, for each $x,y\in G,\,x\rho y$ implies $f(x)\rho f(y)$. As is well-known, there is a lattice isomorphism between the congruence lattice, $\text {Con}(G)$, of congruences on *G* and the lattice, $\eta (G)$, of normal subgroups of *G*. Thus $f\in M_0 (G)$ is congruence preserving if *f* is compatible with every normal subgroup of *G*. That is for $x,y\in G$ we have $f\in C_0 (G)$ if and only if $x+H=y+H$ implies $f(x)+H=f(y)+H$, for each $H\in \eta (G)$.

Recall for any subgroup *H* of *G* the *normal closure* of *H* in *G*, denoted by $\overline{H}$, is defined by $\overline{H}=\bigcap \{N\unlhd G\mid H \subseteq N\}$. For $x\in G$ we write $\overline{x}$ for $\overline{\langle x\rangle }$, the principal closure of *x*. Thus we get the following characterization of congruence preserving functions: Let $f\in M_0 (G)$. Then $f\in C_0 (G)$ if and only if $f(x)-f(y)\in \overline{x-y}$ for each $x,y\in G$.

Using the above definitions one finds that every zero fixing unary polynomial on *G* is congruence preserving so $I(G)=P_0 (G)\subseteq C_0 (G)\subseteq M_0 (G)$. If every congruence preserving function is also a polynomial then the group *G* is said to be *1-affine complete*. Finite abelian 1-affine complete groups have been characterized by Nöbauer [14]. All 1-affine complete groups of order up to 100 can be found in [17]. In relation to our problem under consideration, $C_0 (G)$ will be a ring if $P_0 (G)$ is a ring and *G* is 1-affine complete.

It is known when $I(G)=P_0 (G)$ is a ring [8]. A group *G* is said to be a 2-Engel group if $[x,[x,y]]=0$ for all $x,y\in G$. Equivalently (see [8, 12, 13]) *G* is a 2-Engel group if every element of *G* commutes with all of its conjugates. A 2-Engel group *G* is nilpotent of class at most 3 and, for finite groups, a 2-Engel group of class 3 must be a group of order 3$^n$. The smallest 2-Engel group of class 3 is the Burnside group, *B*(3, 3), of order 3$^7$.

### Theorem 1.1

(Chandy) The near-ring *I*(*G*) is a ring if and only if *G* is a 2-Engel group. Moreover, *I*(*G*) is a commutative ring if and only if *G* is of class 2.

In the remainder of this paper we restrict to finite groups *G*. In the next section we first show when considering $C_0 (G)$ we may restrict to *p*-groups, *p* a prime. Using results of Nöbauer [14] we completely characterize those finite abelian groups *G* such that $C_0 (G)$ is a ring.

In Sect. 3 we turn to nonabelian groups and find several necessary conditions for $C_0 (G)$ to be a ring. In this section we focus on certain properties of the lattice $\eta (G)$ of normal subgroups. In the final section we consider internal properties of the group *G* and we conclude with a complete answer to our question for groups of order $p^n ,\,1\le n\le 5,\,p>2$.

## The abelian case

We begin this section by showing that, for finite groups, we can restrict to *p*-groups, where as usual *p* denotes a prime integer. We use results of Nöbauer, [14], to obtain this restriction.

We recall from [14] that a direct sum $G=G_1 \oplus \cdots \oplus G_n$ is said to be *skew-free* if every congruence $\rho$ of *G* is of the form $\rho =\rho _1 +\cdots +\rho _n$ where the $\rho _i$ are congruences on the $G_i ,\,i=1,2,\dots ,n$. In particular when *G* is a finite nilpotent group with direct sum of its unique Sylow subgroups $G=S_{p_1}(G)\oplus \cdots \oplus S_{p_n}(G)$ then *G* is skew-free. Recall from the introduction that $\text {Con}(G)$ is lattice isomorphic to $\eta (G)$, the lattice of normal subgroups of *G*.

### Theorem 2.1

[14, Satz 1] Let $G=A\oplus B$ be skew-free. Then the map $\psi : C_0(G) \rightarrow C_0(A)+C_0(B)$ given by $\psi (\rho )=(\rho _A,\rho _B)$, $\rho \in C_0(G)$ is a near-ring isomorphism.

From straight forward calculations one finds the following theorem and corollary.

### Theorem 2.2

Let *G* be a finite nilpotent group, $G=S_{p_1}(G)\oplus \cdots \oplus S_{p_n}(G)$. Then $C_0 (G)\cong C_0 (S_{p_1}(G))\oplus \cdots \oplus C_0 (S_{p_n}(G))$.

### Corollary 2.3

Let *G* be a finite group with Sylow decomposition as in Theorem 2.2. Then $C_0 (G)$ is a ring if and only if $C_0 (S_{p_i}(G))$ is a ring for each $i\in \{1,2,\dots ,n\}$.

Hence we only need to consider groups, *G*, of prime power order when investigating the structure of the near-ring, $C_0 (G)$, of zero fixing congruence preserving functions on *G*.

We now turn to abelian groups. We need a further result of Nöbauer [14].

### Theorem 2.4

[14, Satz 3,4] Let *p* be a prime, $\mathbb {Z}_{p^\alpha }$ be the cyclic group of order $p^\alpha$ and let $A=\mathbb {Z}_{p^{\alpha _1}} \oplus \cdots \oplus \mathbb {Z}_{p^{\alpha _n}}$ with $\alpha _1 \ge \alpha _2 \ge \cdots \ge \alpha _n$. Then *A* is 1-affine complete if and only if one of the following conditions holds:

- $$n>1,\,\alpha _1 =\alpha _2 ,\,p$$ arbitrary,
- $$n>1,\,\alpha _1 =\alpha _2 +1,\,p=2$$ or
- $$n=1,\,\alpha _1 =1,\,p=2$$.

### Theorem 2.5

Let *A* be an abelian group of prime power order. The following are equivalent:

1. *A* is 1-affine complete;
2. $$C_0 (A)$$ is a ring;
3. $C_0 (A)$ is a commutative ring.

### Proof

Since *A* is abelian, *A* is a 2-Engel group of nilpotency class at most 2 so $I(A)=P_0 (A)$ is a commutative ring. If *A* is 1-affine complete then $C_0 (A)$ is a ring. Thus (1) implies (3).

It is clear that (3) implies (2) so it remains to show (2) implies (1). We assume *A* is not 1-affine complete and show $C_0 (A)$ is not a ring. From Theorem 2.4 we know the form *A* has to be so that *A* is not 1-affine complete.

Case (i) $|A|=p^m$ for some prime $p>2$. In this case we must have $A\cong \mathbb {Z}_{p^{\alpha _1}} \oplus \cdots \oplus \mathbb {Z}_{p^{\alpha _n}}$ with $n=1$ or $\alpha _1 >\alpha _2 \ge \cdots \ge \alpha _n$. We let $\mathbb {Z}_{p^{\alpha _1}} =\langle g\rangle$ and let $D=\{x\in A\mid p^{\alpha _1 -1}x=0\}=\{(a_1 ,\dots ,a_n )\mid a_1 \in \langle pg\rangle \}$. Define $c:A\rightarrow A$ by
  $$\begin{aligned} c(x)= {\left\{ \begin{array}{ll} (0,\dots ,0), &{} x\in D\\ (p^{\alpha _1 -1}g,0,\dots ,0), &{} x\notin D. \end{array}\right. } \end{aligned}$$
  We show $c\in C_0 (A)$. To this end, let $x,y\in A$. If $x,y\in D$ or $x,y\notin D$ then $c(x)-c(y)=(0,\dots ,0)\in \overline{x-y}$ so we take $x\notin D,\,y\in D$. Thus $c(x)-c(y)=(p^{\alpha -1}g,0,\dots ,0)$. Now $p^{\alpha _1 -1}(x-y)=(p^{\alpha _1 -1}kg,0,\dots ,0),\,p\not \mid k$ so $x\notin D$. But then $(p^{\alpha _1 -1}g,0,\dots ,0)\in \overline{x-y}$ so $c\in C_0 (A)$. Further, $[c(\text {id}+\text {id})]((g,0,\dots ,0))=c(\text {id}((g,0,\dots ,0))+\text {id}((g,0,\dots ,0)))=c((2g,0,\dots ,0))=(p^{\alpha _1 -1}g,0,\dots ,0)$ while $[c\circ \text {id}+c\circ \text {id}]((g,0,\dots ,0))=2c((g,0,\dots ,0))=2(p^{\alpha _1-1}g,0,\dots ,0)\ne (p^{\alpha _1-1}g,0,\dots ,0)$. Hence $C_0(G)$ is not a ring.
Case (ii) $|A|=2^m$. In this case $A=\mathbb {Z}_{2^{\alpha _1}}\oplus \cdots \oplus \mathbb {Z}_{2^{\alpha _n}}$ with either $n>1$ and $\alpha _1 -1>\alpha _2 \ge \cdots \ge \alpha _n$ or $n=1$ and $\alpha _1 >1$. Again we handle both cases together and as above we let $\mathbb {Z}_{2^{\alpha _1}}=\langle g\rangle$. Let $D=\{x\in A\mid 2^{\alpha _1 -2}x=0\}=\{(a_1 ,\dots ,a_n )\mid a_1 \in \langle 4g\rangle \}$ and further let $\bar{D}=D\cup (D+(2g,0,\dots ,0))$. Define $c:A\rightarrow A$ by
  $$\begin{aligned} c(x)= {\left\{ \begin{array}{ll} (0,0,\dots ,0), &{} x\in \bar{D}\\ (2^{\alpha _1 -2}g,0,\dots ,0), &{} x\notin \bar{D}. \end{array}\right. } \end{aligned}$$
  As above we show $c\in C_0 (A)$. Let $x,y\in A,\,x\notin \bar{D},\,y\in \bar{D}$. Then $c(x)-c(y)=(2^{\alpha _1 -2}g,0,\dots ,0)$. Now since $x\notin \bar{D},\,x=(kg,0,\dots ,0)$ where $k\in \{1,3\}$. Thus $2^{\alpha _1 -2}(x-y)=(2^{\alpha _1 -2}kg,0,\dots ,0)$ so $(2^{\alpha _1 -2}g,0,\dots ,0)\in \overline{x-y}$. Using $f=\text {id},\,h=3\cdot \text {id}$ we have $[c(f+g)]((g,0,\dots ,0)) = [c(\text {id}+3\cdot \text {id})]((g,0,\dots ,0))=c(\text {id}((g,0,\dots ,0))+3\cdot \text {id}((g,0,\dots ,0)))=c((4g,0,\dots ,0))=(0,\dots ,0)\ne c((g,0,\dots ,0))+c((3g,0,\dots ,0))$. So $C_0 (A)$ is not a ring. $\square$

We now have a characterization of those finite abelian groups *A* for which $C_0 (A)$ is a ring.

### Corollary 2.6

Let *A* be a finite abelian group. The following are equivalent:

1. *A* is 1-affine complete;
2. $$C_0 (A)$$ is a ring;
3. $C_0 (A)$ is a commutative ring.

### Proof

As in Theorem 2.5 (1) implies (3) and (3) implies (2). Using the Sylow decomposition of *A* we see that (2) implies (1) follows from Theorem 2.5 and Nöbauer [14, Lemma 5]. $\square$

In the next section we give several necessary conditions for $C_0 (G)$ to be a ring.

## Lattice conditions

We start with some conditions on the congruence lattice, $\text {Con}(G)$. Since $\text {Con}(G)$ is lattice isomorphic to the normal subgroup lattice, $\eta (G)$, we often state our properties in terms of normal subgroups.

We recall that *G* must be a 2-Engel group, and thus nilpotent of class at most 3, for $C_0 (G)$ to be a ring.

Our first lattice concept is that of splitting pair. This property has been used previously [3, 5, 15]. Let $D,E\in \eta (G),\,D\subset G,\,\{0\}\subset E$. The pair (*D*, *E*) is called a *splitting pair* if for each $N\in \eta (G),\,N \subseteq D$ or $N \supseteq E$. If *G* has a splitting pair then *G* *splits*.

Now let (*D*, *E*) be a splitting pair for *G* and let $0\ne b\in E$. Define $f:G\rightarrow G$ by

$$\begin{aligned} f(x)= {\left\{ \begin{array}{ll} 0, &{} x\in D\\ b, &{} \text {otherwise.} \end{array}\right. } \end{aligned}$$

We show $f\in C_0 (G)$. Let $x,y\in G$ and let $H\in \eta (G)$ with $x+H=y+H$. If $E\subseteq H$ then since $f(x)-f(y) \in \{-b,0,b\} \subseteq E$ we get $f(x)+H=f(y)+H$. If $H\subseteq D$ and $x\in D$ then $x+H\subseteq D$ and so $y+H\subseteq D$ which means $y\in D$. By symmetry if $x\notin D$ then $y\notin D$, hence in both cases $f(x)+H=f(y)+H$. This establishes that $f\in C_0 (G)$. Now if $C_0 (G)$ is a ring then for $v\notin D,\,[f\circ (\text {id}+\text {id})](v)=[(f\circ \text {id}+f\circ \text {id})](v)$ or $f(2v)=2f(v)$. If $2v \notin D$ then $b=2b$, which contradicts $b\ne 0$. Therefore $2v \in D$ and further $0=2b$. Since $0\ne b$ was arbitrary in *E* we get $E\cong (\mathbb {Z}_2 )^n$ for some $n>0$. This establishes:

### Theorem 3.1

Let *G* be a finite group such that $2\not \mid | G|$. If *G* splits then $C_0 (G)$ is not a ring.

### Proof

From the above discussion, when *G* splits then *G* has a subgroup $E\cong (\mathbb {Z}_2 )^n ,\,n>0$ which is a contradiction since $2\not \mid | G|$. $\square$

In particular if *G* is a finite *p*-group, $p>2$, and *G* splits, then $C_0 (G)$ is not a ring. The situation is different in the non-split case as the next examples illustrate. These examples and some of the calculations have been done with GAP using the package Sonata [2].

### Example 3.2

1. Group with GAP index $3^7 /6010$. $G=\langle e_1 ,e_2 ,e_3 ,e_4 ,c_1 ,c_2 ,c_3 \rangle ,\,3e_i =3c_j =0,\,[e_i ,c_j ]=[c_k ,c_j ]=0,\,[e_1 ,e_2 ]=c_1 ,\,[e_1 ,e_3 ]=c_2 ,\,[e_2 ,e_3 ]=c_3$ otherwise $[e_l ,e_m ]=0,\,i=1,2,3,4,\,j,k=1,2,3$. Thus *G* is a group of exponent 3, nilpotent of class 2 with $G^\prime =\langle c_1 ,c_2 ,c_3 \rangle \subseteq Z(G)$ [9]. From GAP, *G* does not split but is 1-affine complete so $C_0 (G)$ is a ring since nilpotent of class 2 means *G* is 2-Engel.
2. GAP index $3^7 /6576$. $G=\langle e_1 ,e_2 ,e_3 ,e_4 ,c_1 ,c_2 ,c_3 \rangle ,\,3e_i =3c_i =0,\,[e_i ,c_j ]=[c_k ,c_j ]=0,\,i=1,2,3,4,\,j,k=1,2,3$ with $[e_1 ,e_2 ]=c_1 ,\,[e_1 ,e_3 ]=[e_2 ,e_4 ]=c_2 ,\,[e_3 ,e_4 ]=c_3$, otherwise $[e_m ,e_l ]=0$. Again *G* is of exponent 3, nilpotent of class 2, with $G^\prime =\langle c_1 ,c_2 ,c_3 \rangle \subseteq Z(G)$ [9]. Using GAP, *G* does not split and is not 1-affine complete. We show $C_0 (G)$ is not a ring. For $x\in G,\,x=\alpha e_1 +\beta e_2 +\gamma e_3 +\delta e_4 +d$ where $d\in G^\prime$. Define $f:G\rightarrow G$ by
  $$\begin{aligned} f(x)= {\left\{ \begin{array}{ll} 2(c_1 +c_2 +c_3 ), &{} \text {if }\beta +\gamma =3,\\ 2c_3 , &{} \text {if }\beta =0,\gamma \ne 0,\\ 2c_1 , &{} \text {if }\beta \ne 0,\gamma =0,\\ 2c_1 +c_2 +2c_3 , &{} \text {if }\beta =\gamma \ne 0,\\ 0, &{} \text {otherwise.} \end{array}\right. } \end{aligned}$$
  Calculations show that $f\in C_0 (G)$. Now $f\circ (\text {id}+\text {id})(e_3 )=f(2e_3 )=2c_3$ while $(f\circ \text {id}+f\circ \text {id})(e_3 )=2c_3 +2c_3 =c_3 \ne 2c_3$. Thus $C_0 (G)$ is not a ring.

Above we denoted the normal closure of $x\in G$ by $\overline{x}$. For $x\in G$, let $P_0 (G)x=\{p(x)\mid p\in P_0 (G)\}$ and $C_0 (G)x=\{c(x)\mid c\in C_0 (G)\}$.

### Lemma 3.3

Let *G* be a group and let $x\in G$.

1. $$P_0 (G)x=\overline{x}$$.
2. $P_0 (G)x=C_0 (G)x$. If further *G* is 2-Engel then
3. $$\overline{x}$$ is abelian;
4. $\langle C_0 (G),+\rangle$ is an abelian group.
5. If *G* is nilpotent of class at most 2 then $\overline{x}=\langle x\rangle +[x,G]$.

### Proof

1. Clearly $P_0 (G)x\subseteq \overline{x}$. On the other hand, $P_0 (G)x$ is a normal subgroup of *G* containing *x*, so $\overline{x}\subseteq P_0 (G)x$.
2. One has $P_0 (G)x\subseteq C_0 (G)x$. For $c\in C_0 (G),\,c(x)-c(0)\in \overline{x-0}$ so $c(x)\in \overline{x}$. Thus $C_0 (G)x\subseteq \overline{x}=P_0 (G)x$.
3. When *G* is 2-Engel, $P_0 (G)$ is a ring so with 1) we get that $P_0 (G)x$ is an abelian group.
4. Follows from 2) since *G* is 2-Engel.
5. In [10] Ecker shows $p\in P_0 (G)$ has the form $p(x)=kx+[x,g]$ for some integer *k* and $g\in G$ when *G* is nilpotent of class at most 2. Thus $P_0 (G)x\subseteq \langle x\rangle +[x,G]$. But $\langle x\rangle +[x,G]\subseteq \overline{x}=P_0 (G)x$. $\square$

We next give a characterization of those groups *G* for which $C_0 (G)$ is a ring. The usefulness of this result is somewhat limited since it requires knowledge of all $c\in C_0 (G)$.

### Theorem 3.4

Let *G* be a finite 2-Engel group. Then $C_0 (G)$ is a ring if and only if $\left. c\right| _{\overline{x}}\in \text {End}(\overline{x})$ for each $c\in C_0 (G)$ and $x\in G$.

### Proof

Let *c*, *f*, *g* be arbitrary in $C_0 (G)$ and let $x\in G$. From Lemma 3.3 $\langle C_0 (G),+\rangle$ is an abelian group. Suppose $\left. c\right| _{\overline{x}}\in \text {End}(\overline{x})$ for each $x\in G$. Then $c\circ (f+g)(x)=c(f(x)+g(x))=c(f(x))+c(g(x))=(c\circ f+c\circ g)(x)$, since $f(x), g(x) \in \bar{x}$ by (1) and (2) of Lemma 3.3. Thus $C_0 (G)$ is a ring.

For the converse let $a,b\in \overline{x}$. Thus by 1) and 2) of Lemma 3.3 there exist $h,l\in C_0 (G),\,a=h(x),\,b=l(x)$. Now let $c\in C_0 (G)$. It is clear that $c(\overline{x})\subseteq \overline{x}$. Moreover since $C_0 (G)$ is a ring we have $c(a+b)=c(h(x)+l(x))=c(h(x))+c(l(x))=c(a)+c(b)$ which shows $c\in \text {End}(\overline{x})$. $\square$

Therefore if one can construct a congruence preserving function that is not linear on some normal closure $\overline{x},\,x\in G$, where *G* is 2-Engel, then $C_0 (G)$ is not a ring. In the next example, using Theorem 3.4, we show that the result of Theorem 3.1 on groups which split is not true for $p=2$.

### Example 3.5

Let *G* be a semidirect product of $\mathbb {Z}_4$ and $\mathbb {Z}_4$: $G=\langle x,y\mid 4x=4y=0,\,y+x=3x+y\rangle$. We have $Z(G)=\langle 2x,2y\rangle$ and one verifies that $D=Z(G)$ and $E=\langle 2x\rangle$ is a splitting pair for $\eta (G)$. Define $c:G\rightarrow G$ by

$$\begin{aligned} c(w)= {\left\{ \begin{array}{ll} 0, &{} w\in Z(G),\\ 2x, &{} w\notin Z(G). \end{array}\right. } \end{aligned}$$

Let $u,v\in G$. If $u,v\in Z(G)$ or $u,v\notin Z(G)$ then $c(u)-c(v)=0\in \overline{u-v}$. If $u\notin Z(G)$ and $v\in Z(G)$ then $u-v\notin Z(G)$ and so $\overline{u-v}\supseteq \langle 2x\rangle$ since $(Z(G),\langle 2x\rangle )$ is a splitting pair. Thus $c(u)-c(x)=2x\in \overline{u-v}$ so $c\in C_0 (G)$ but $c\notin P_0 (G)$ since $c(x)=2x$ while $c(y)=2x\ne 2y$.

Using GAP one finds $| P_0 (G)|=16$ and $| C_0 (G)|=32$ so we have $C_0 (G)=P_0 (G)+\langle c\rangle =\{p+lc\mid l\in \{0,1\},\,p\in P_0 (G)\}$. For $w\notin Z(G)$, calculations show that *c* is linear on $\overline{w}$. Thus for all $w\in G,\,\left. c\right| _{\overline{w}}\in \text {End}(\overline{w})$. Thus for each $p\in P_0 (G),\,p+c$ is linear on each $\overline{w}$ so from Theorem 3.4 $C_0 (G)$ is a ring.

We turn to another lattice condition, a particular case of a splitting pair. If (*D*, *E*) is a splitting pair for $\eta (G)$ and $D=E$ we say *D* is a *cutting element* and *G* *cuts*.

### Lemma 3.6

Let *G* be a finite *p*-group of nilpotency class at most 2 such that *I* is a cutting element for $\eta (G)$. Then $I\subseteq Z(G)$.

### Proof

Let *T* be the maximal cutting element for $\eta (G)$ which exists since *G* is finite and cutting elements form a chain in $\eta (G)$. We have $T\supseteq I$. If *G* is abelian then $I\subseteq Z(G)=G$ so we take *G* of class 2, hence $G^\prime \subseteq Z(G)$. If *T* is also a maximal element in $\eta (G)$ then *G* has a unique maximal normal subgroup. Thus from [16], *G* is cyclic, contrary to *G* being of class 2. Thus we suppose *T* is not a maximal element in $\eta (G)$. If $T\subseteq G^\prime$ then *T*, hence *I*, is contained in *Z*(*G*). To complete the proof we show $G^\prime \subset T$ cannot occur. Suppose $G^\prime \subset T$ and let $N\in \eta (G)$ be maximal with $G^\prime \subseteq N \subset T$. Since $G/G^\prime$ is abelian, *G* / *N* is also abelian. Therefore *G* / *N* has a unique minimal normal subgroup *T* / *N*. But this means that *G* / *N* is subdirectly irreducible and (from [7] p. 64) *G* / *N* is cyclic. However this contradicts the fact that *T* is the unique maximal cutting element but not a maximal element in $\eta (G)$. Thus we have $I\subseteq Z(G)$. $\square$

### Theorem 3.7

Let *G* be a finite nonabelian *p*-group such that *G* cuts. Then $C_0 (G)$ is not a ring.

### Proof

Let *I* be a cutting element. If *G* is of nilpotency class greater than 3 then *G* is not 2-Engel, hence $C_0 (G)$ is not a ring. Further if $p>2$ then from Theorem 3.1, $C_0 (G)$ is not a ring. Therefore $p=2$ and *G* is nilpotent of class 2, $| G|=2^n ,\,n\ge 3$. From Lemma 3.6 we get $I\subseteq Z(G)$.

Let $T_1$ be a transversal of *G* / *I* with $0\in T_1$. Let $t\in T_1 -\{0\}$ and define $T_2 =(T_1 \backslash (t+t+I))\cup \{t+t\}$. We note $T_2$ is a transversal of *G* / *I* with $\{t,t+t\}\subseteq T_2$. Suppose first that $t+t\notin I$ so $0\in T_2$. Let $0\ne e$ be in *I* and define $h:G\rightarrow G$ by

$$\begin{aligned} h(x)= {\left\{ \begin{array}{ll} e, &{} x\in t+t+I,\\ 0, &{} \text {otherwise.} \end{array}\right. } \end{aligned}$$

We note that $h(0)=0$. To show $h\in C_0 (G)$ it suffices to show for $r\in T_2 -\{t+t\},\,d_1 ,d_2 \in I$ that $h(t+t+d_1 )-h(r+d_2 )\in \overline{t+t+d_1 -d_2 -r}$, that is, we must show $e\in \overline{t+t+d_1 -d_2 -r}$. We first observe that $t+t+d_1 -d_2 -r=t+t-r+d_1 -d_2 \notin I$ since $r\in T_2 -\{t+t\}$. Therefore $\overline{t+t+r+d_1 -d_2}\nsubseteq I$ and, since *I* cuts $\eta (G),\,I\subseteq \overline{t+t+r+d_1 -d_2}$ giving the desired result that $h\in C_0 (G)$. But $h(t+t)=e\ne h(t)+h(t)$. Since *h* is not linear on $\overline{t},\,C_0 (G)$ is not a ring.

Suppose next we have $0\ne t+t\in I$. Using $T_1$ we define $f:G\rightarrow G$ by $f(x)=j$ where $x=r+j,\,r\in T_1 ,\,j\in I$. Since $0\in T_1 ,\,f(0)=0$. For $x=r_1 +j_1 ,\,y=r_2 +j_2 ,\,f(x)-f(y)=j_1 -j_2$. If $r_1 =r_2 ,\,j_1 -j_2 \in \overline{r_1 -r_2 +j_1 -j_2}$. If $r_1 \ne r_2$ then $r_1 -r_2 +j_1 -j_2 \notin I$ so $I\subseteq \overline{r_1 -r_2 +j_1 -j_2}$, hence $f(x)-f(y)=j_1 -j_2 \in \overline{x-y}$. Thus $f\in C_0 (G)$. Since $t+t\in I,\,t+t=0+t+t$ which means $f(t+t)=t+t$. But $t=t+0$ so $f(t)=0=f(t)+f(t)\ne f(t+t)$. This shows that $C_0 (G)$ is not a ring.

For the final case we have $t+t\in I$ and $t+t=0$. Define $l:G\rightarrow G$ by

$$\begin{aligned} l(x)= {\left\{ \begin{array}{ll} 0, &{} x\in I,\\ j, &{} x\notin I\text { and }x=r+j,\,r\in T_2 =T_1 . \end{array}\right. } \end{aligned}$$

For $x\notin I,\,y\in I$ say $x=r+j,\,r\in T_2 ,\,j\in I$ we have $l(x)-l(y)=j$. Moreover $I\subseteq \overline{r+j-y}$ since $r+j-y\notin I$. Thus $l\in C_0 (G)$. Since $t+t\in I$ for any $0\ne i\in I,\,l(t+t+i)=0$. Further since $t\notin I,\,I\subseteq \overline{t}$, hence $t+i\in \overline{t}$. Now $l(t)+l(t+i)=0+i\ne 0$ so *l* is not linear on $\overline{t}$. Thus in all cases we have found when *G* is cut, $C_0 (G)$ is not a ring. $\square$

## Structural conditions

In this section we focus on group theoretical properties of a group *G* to determine when $C_0 (G)$ is a ring. We restrict to nilpotency class 2 and *p*-groups $p\ge 3$.

### Theorem 4.1

Let *G* be a nonabelian *p*-group, $p>2$ such that $G^\prime$ is cyclic. Then $C_0 (G)$ is not a ring.

### Proof

Let $x\in G-Z(G)$. Thus $\{0\}\ne [x,G]\subseteq [G,G]$. By hypothesis, $G^\prime =\langle r\rangle$ for some $r\in G$ so we have $\overline{r}=\langle r\rangle =G^\prime$.

Let $\langle r^\prime \rangle$ be the unique subgroup of order *p* in $G^\prime$. Since [*x*, *G*] is a nonzero cyclic subgroup of $G^\prime$, $[x, G^\prime ]$ contains a cyclic subgroup of order *p* of $G^\prime$, hence we have $\langle r^\prime \rangle$ is a subgroup of $[x,G^\prime ]$. Moreover, for each $g\in G,\,p(-g+r^\prime +g)=0$ so $-g+r^\prime +g$ is in $\langle r^\prime \rangle$ which in turn leads to the fact that $\langle r^\prime \rangle$ is a normal subgroup of [*x*, *G*]. Therefore, for $x\in G-Z(G)$, $\overline{r^\prime }=\langle r^\prime \rangle \subseteq [x,G]\subseteq \overline{x}$.

Define $$h:G\rightarrow G$$ by

$$\begin{aligned} h(x)= {\left\{ \begin{array}{ll} r^\prime , &{} x\notin Z(G),\\ 0, &{} x\in Z(G). \end{array}\right. } \end{aligned}$$

We show $h\in C_0 (G)$. To this end let $u\notin Z(G),\,v\in Z(G)$. Then $h(u)-h(v)=r^\prime$ which is in $\overline{u-v}$ since $u-v\notin Z(G)$. This gives $h\in C_0 (G)$. For $w\notin Z(G),\,2w=w+w\notin Z(G)$ since $p>2$ so if $2w \in Z(G)$, we would have $w \in Z(G)$, a contradiction. From this observation, $h(w+w)=r^\prime \ne 2r^\prime =h(w)+h(w)$. Therefore $C_0 (G)$ is not a ring. $\square$

We actually have a little more.

### Corollary 4.2

If *G* is a nonabelian *p*-group, $p>2$, such that $G^\prime$ is cyclic, then $\eta (G)$ splits.

### Proof

From the proof of Theorem 4.1 we get if $N\trianglelefteq G$ and $N\nsubseteq Z(G)$ then for $x\in N-Z(G),\,N\supseteq \overline{x}\supseteq \overline{r^\prime }$. Thus $\langle Z(G),\overline{r^\prime }\rangle$ is a splitting pair for $\eta (G)$. $\square$

### Corollary 4.3

Let *G* be a *p*-group, $p>2$, of nilpotency class 2. If *G* is 2-generated (generated by 2 elements) then $C_0 (G)$ is not a ring.

### Proof

If $G=\langle x,y\rangle$ then one finds $G^\prime =\langle [x,y]\rangle$ so $G^\prime$ is cyclic [6]. The result now follows from the above theorem. $\square$

We remark that Example 3.5 shows that Corollary 4.3 does not hold for $p=2$.

Recall that a group *G* is abelian by cyclic, or *G* is said to be an extension of an abelian group by a cyclic group if there exists an abelian normal subgroup *A* of *G* such that *G* / *A* is cyclic. For finite *G* one may always take *A* to be a maximal abelian normal subgroup.

### Theorem 4.4

Let *G* be a nonabelian *p*-group, $p>2$, of nilpotency class 2 which is abelian by cyclic. Then $C_0 (G)$ is not a ring.

### Proof

We let *A* be a maximal abelian normal subgroup such that ${G}/{A}\cong \mathbb {Z}_{p^k},\,k$ a positive integer. Let ${G}/{A}=\langle b+A\rangle$ so $G=\langle A,b\rangle$. Since *G* is nonabelian there exists $a_1 \in A$ such that $[a_1 ,b]\ne 0$. Every $x \in G$ can be decomposed into a sum of the form $x=a+\beta b+c$, with $a\in A,\, \beta \in \mathbb {Z},\,c\in G^\prime$. (Recall the basic assumption that *G* is of class 2 so $[G,G]\subseteq Z(G)$.)

Using this decomposition we define $f:G\rightarrow G$ by

$$\begin{aligned} f(x)= {\left\{ \begin{array}{ll} [b,a_1], &{} p\not \mid \beta ,\\ 0, &{} p\mid \beta . \end{array}\right. } \end{aligned}$$

Let $x \in G$. We note $[x,a_1 ]=\beta [b,a_1 ]$ so when $p\not \mid \beta ,\,[b,a_1 ]\in \overline{x}$. Also, $f\in C_0 (G)$. For if $u,v\in G,\,u=a+\beta b+c,\,v=a^\prime +\beta ^\prime b+c^\prime ,\,a,a^\prime \in A,\,\beta ,\beta ^\prime \in \mathbb {Z},\,c,c^\prime \in [G,G]$ with $p\not \mid \beta$ and $p\mid \beta ^\prime$ then $p\not \mid (\beta -\beta ^\prime )$ so $[b,a_1]\in \overline{u-v}$. Thus $f(u)-f(v)=[b,a_1]\in \overline{u-v}$. For $u=a+\beta b+c$ with $p\not \mid \beta ,\,f(u)+f(u)=[b,a_1 ]+[b,a_1 ]$ while $f(u+u)=f(2u)=[b,a_1 ]$. Using Theorem 3.4, $C_o (G)$ is not a ring. $\square$

### Corollary 4.5

Let *G* be a nonabelian *p*-group, $p>2$, of class 2 such that there exists $g\in G$ with ${G}/{C_G (g)}$ cyclic. Then $C_0 (G)$ is not a ring.

### Proof

Let ${G}/{C_G (g)}=\langle b+C_G (g)\rangle$ so $G=\langle C_G (g),b\rangle$. For $x\in G,\,x=w+\beta b+c,\,w\in C_G (g),\,\beta \in \mathbb {Z},\,c\in G^\prime$ and since $b\notin C_G (g),\,[b,g]\ne 0$. Now $[x,g]=\beta [b,g]$. The remainder of the proof is as above and is omitted. $\square$

As we did following Theorem 4.1, we again show that under the hypothesis of Theorem 4.4, $\eta (G)$ splits.

### Theorem 4.6

Let *G* be a *p*-group, nilpotent of class 2, which is abelian by cyclic. Then $\eta (G)$ splits.

### Proof

As above we let *A* be a maximal abelian normal subgroup with ${G}/{A}=\langle b+A\rangle$. Then $G=\langle A,b\rangle$ and $b\notin A$ so there exists $a_1 \in A,\,[a_1 ,b]\ne 0$. Let $A=\langle a_1 ,a_2 ,\dots ,a_n \rangle$ so $G=\langle a_1 ,\dots ,a_n ,b\rangle$. Let $A_0 =\langle a_1 ,\dots ,a_n ,pb\rangle$. If $pb=0$ then $A_0 =A$ and the same type of argument works. We first show that $A_0$ is a normal subgroup of *G*. Let $g=a+\beta b+c\in G,\,a\in A,\,\beta \in \mathbb {Z},\,c\in G^\prime$. It suffices to show $-g+pb+g\in A_0$. To this end, $-g+pb+g=-c-\beta b-a+pb+a+\beta b+c=-\beta b-a+pb+a+\beta b=-\beta b+pb+[pb,a]+\beta b=pb+[pb,a]\in A_0$. Therefore $A_0 \trianglelefteq G$.

Further $A_0 \subset G$. For if $A_0 =G$ then $b\in A_0=\langle a_1, a_2, \dots , a_n, pb\rangle$. From this, $b=a+\alpha (pb)+c$ where $a \in A$ and *c* is a sum of commutators. Since *A* is a maximal abelian normal subgroup we have $Z(G) \subseteq A$ and since *G* is nilpotent of class 2, $[G,G] \subseteq Z(G) \subseteq A$. Thus $c\in A$. Hence $(1-\alpha p)b\in A$ which implies $b \in A$ since $1-\alpha p$ is invertible modulo *p*, a contradiction. Thus $A_0 \ne G$.

Now let $N\in \eta (G)$ such that $N\nsubseteq A_0$. For $n\in N-A_0 ,\,n=a+\delta b+c ,\,a\in A,\,p\not \mid \delta ,\,c\in G^\prime$, hence $[n,a_1 ]=\delta [b,a_1 ]$ and since $p\not \mid \delta ,\,0\ne [b,a_1 ]\in \overline{n}\subseteq N$. From this, $\langle [b,a_1 ]\rangle \subseteq N$ for each $N\in \eta (G)$ such $N\nsubseteq A_0$. This shows that $(A_0 ,\langle [b,a_1 ]\rangle )$ is a splitting pair for $\eta (G)$. $\square$

In Theorems 4.6 and 4.1 one has the situation where *G* has a partition $G=X\cup (G-X)$ with the property that $\bigcap \{ \overline{u} \mid u \in X\} \ne \{0\}$ and, for each $u\in X$, for each $v\in G-X,\,u-v\in X$. It is an open question if this condition implies the splitting of $\eta (G)$.

We apply the above results to *p*-groups, $p>2$, of small order. Let *G* be a group of order $p^n ,\,p>2,\,1\le n\le 5$. When $n=1$ or $n=2$, *G* is abelian so $C_0 (G)$ is a ring if and only if $G\cong \mathbb {Z}_p +\mathbb {Z}_p$. Thus we take $n\ge 3$ and since the abelian case is known from Theorems 2.4 and 2.5 we restrict to nonabelian groups.

### Theorem 4.7

Let *G* be a nonabelian *p*-group, $p>2$ of order $p^n ,\,3\le n\le 5$ such that *G* is nilpotent of class 2. Then $C_0 (G)$ is not a ring.

### Proof

(i) $n=3.$ Since *g* is nonabelian we have $| Z(G)|=p$. Thus $\{0\}\ne G^\prime \subseteq Z(G)$, hence $G^\prime$ is cyclic and the result follows from Theorem 4.1.
(ii) $n=4.$ Let $\Phi (G)$ denote the Frattini subgroup of *G*. We know $G^\prime \subseteq \Phi (G)$ and if $|{G}/{\Phi (G)}|=p^k$ then *G* is generated by *k* elements [16]. If $|\Phi (G)|=p$ then $G^\prime$ is cyclic while if $|\Phi (G)|=p^2$ then *G* is generated by 2 elements. Using Theorem 4.1 and Corollary 4.3 we see that $C_0 (G)$ is not a ring. If $|\Phi (G)|=p^3$ then *G* has a unique maximal normal subgroup which cuts *G*. The result now follows from Theorem 3.7.
(iii) $n=5$. If $|\Phi (G)|=p$ or $p^3$ or $p^4$ then as in the above case we have $C_0 (G)$ is not a ring. It remains to consider $|\Phi (G)|=p^2$. We must have $G^\prime =\Phi (G)$ for if $G^\prime \ne \Phi (G)$ then $G^\prime$ is cyclic and we are finished. Thus we have $G^\prime =\Phi (G)\subseteq Z(G)$ since *G* is of class 2. For $x\in G-Z(G)$ define $\varphi _x :G\rightarrow G$ by $\varphi _x (w)=[x,w]$. Since *G* is of class 2, $\varphi _x$ is an endomorphism of *G* and $\ker \varphi _x =C_G (x)\supseteq \langle x\rangle +Z(G)$ while $Im\varphi _x =[x,G]\subseteq \overline{x}$. We have $|\langle x\rangle +Z(G)|=p|Z(G)|$ so if $G^\prime \subset Z(G)$ then $|Z(G)|=p^3$ and $|\ker \varphi _x |=|C_G (x)|=|\langle x\rangle +Z(G)|=p^4$. But this means *G* is abelian by cyclic so the result follows from Theorem 4.4. Thus we take $|G^\prime |=|\Phi (G)|=|Z(G)|=p^2$. Thus $|\ker \varphi _x |=|C_G (x)|\ge |\langle x\rangle +Z(G)|=p^3$. If $|C_G (x)|=p^4$ then the result follows from Corllary 4.5, so we take $|\ker \varphi _x |=|C_G (x)|=p^3$. But then $|Im\varphi _x |=|{G}/{\ker \varphi _x}|=p^2$. Thus $|[x,G]|=p^2$, so $[x,G]=G^\prime$ which means $G^\prime \subseteq \overline{x}$ for each $x\notin Z(G)$. Thus, if $N\trianglelefteq G$ and $N\nsubseteq Z(G)$ then $N\supseteq G^\prime$. This shows that *Z*(*G*) cuts $\eta (G)$ and so $C_0 (G)$ is not a ring. $\square$

$$\square $$

In conclusion we have found that when *G* is a finite abelian *p*-group then $C_0 (G)$ is a ring if and only if *G* is 1-affine complete. For nonabelian *p*-groups, $p=2$, we have seen that $C_0 (G)$ can be a ring properly containing $P_0 (G)$. For $p>2$ we have several classes for which $C_0 (G)$ is not a ring. In fact, for $p>2$ the authors have no example of a nonabelian *p*-group for which $C_0 (G)$ is a ring unless *G* is 1-affine complete. We thus close with the following.

### Conjecture

For finite nonabelian *p*-groups $G,\,p>2,\,C_0 (G)$ is a ring if and only if *G* is 1-affine complete.
